# Huangqi Shengmai Yin Protects against Radiation-Induced Cardiac Fibrosis Injury by Regulating the TGF-*β*1/Smads and MMPs

**DOI:** 10.1155/2019/1358469

**Published:** 2019-05-13

**Authors:** Jing Gu, Yongqi Liu, Hongyan Wu, Hailong Li, Kai Liu

**Affiliations:** ^1^Institute of Integrated Traditional Chinese and Western Medicine, Gansu University of Traditional Chinese Medicine, Lanzhou 730000, China; ^2^Key Laboratory for Transfer of Dunhuang Medicine at the Provincial and Ministerial Level, Gansu University of Traditional Chinese Medicine, Lanzhou 730000, China; ^3^Gansu University of Traditional Chinese Medicine, Lanzhou 730000, China

## Abstract

**Background:**

Radiation-induced heart damage is considered to be a progressive process of fibrosis. Emerging evidence has indicated that the Smads and matrix metalloproteinases (MMPs)/tissue inhibitors of MMPs (TIMP) may be involved in radiation-induced cardiac fibrosis (RICF) by regulating the activation of TGF-*β*1 signaling pathway. Based on this, the present study was undertaken to characterize the effect of Huangqi Shengmai Yin (HSY) on RICF in a rat model.

**Methods:**

Precardiac region of rats was irradiated with 25 Gy X-rays, and their myocardial pathology scores in terms of injury and collagen volume fraction (CVF) and the expression levels of fibrotic molecules were detected.

**Results:**

The pathology scores and CVF in myocardial tissue increased after irradiation, and the expression of* TGF-β1, Col1, *and* Col3* increased. This change indicated that such irradiation promoted the fibrosis damage in rat hearts. The damage was accompanied by an increase in the expression of Smad 2, Smad3, Smad4, and MMP14 and a decrease in the expression of Smad 7 and TIMP1. Administration of HSY weakened the RICF by decreasing pathology score and CVF and decreased the expression of* TGF-β1, Col1, *and* Col3* in irradiated rat hearts. In addition, Smad2, Smad3, Smad4, and MMP14 were downregulated, while Smad 7 and TIMP1 were upregulated during intervention with HSY.

**Conclusions:**

The involvement of the* TGF-β1/Smads *and* MMPs/TIMP* system in RICF was confirmed. This study demonstrated, for the first time, that HSY attenuates the effects of RICF in a rat model. The effect HSY was found to be closely related to the* TGF-β1*/Smads signaling pathway and MMPs system. These results suggest that HSY is a prospective drug for clinical treatment of RICF.

## 1. Introduction

Radiotherapy treatment for thoracic and abdominal tumors can affect the heart which locates in the mediastinum and cause radiation-induced heart damage (RIHD) [1]. Remarkably, the incidence of RIHD has been increasing and is becoming a serious topic of research. In a study published in* Radiotherapy Oncology* in 2015, Cella pointed out that “radioactive heart injury is already as important as the conventional radiative pulmonary fibrosis injury” [2]. However, the pathogenesis of RIHD remains unclear and the disease lacks effective interventions.

RIHD is described as an outside-in, progressive process of fibrosis [3], i.e., radiation-induced cardiac fibrosis (RICF). RICF is mainly characterized by an increase in cardiac fibroblasts (CFs) and increase in the synthesis of myocardial collagen [4]. TGF-*β*1, which is synthesized by CFs, is closely related to fibrosis in the kidneys, liver, lungs, and other important organs after irradiation [5]. It has also been reported that TGF-*β*1 participates in RICF [6]. In previous study, we used PCR array to find that the Smads and MMPs system may be involved in the RICF by regulating the activation of TGF-*β*1 signaling pathway [7]. However, these molecular signaling mechanisms are not well understood in RICF. In traditional Chinese medicine (TCM), radiation is considered to be an evil of heat and poison, with irradiation to the heart causing toxic heat and blood stasis syndrome, deficiency of both Qi and Yin, Qi stagnation, and blood stasis. Therefore, TCM intervention should focus on “supplementing Qi and nourishing Yin and replenishing Yin and nourishing blood”. Shengmai Yin first appeared in Sun Simiao's ‘Qian Jin Yao Fang' in Tang dynasty. Shengmai Yin is composed of ginseng, liriope, and* Schisandra chinensis* and is mainly used for the treatment of coronary heart disease with deficiencies of both Qi and Yin. Chinese astragalus is a holy medicine for nourishing Qi. Additionally, previous studies have found that astragalus can regulate the expression of fibrosis-related molecule in irradiated cardiac fibroblasts (CFs) [7]. In this study, based on the TGF-*β*1/Smads and MMPs, we explored the effects and mechanisms of Huangqi Shengmai Yin (adding astragalus to the prescription of Shengmai Yin to form the Huangqi Shengmai Yin decoction, HSY) on a RICF rat model. This experimentation provided an evidence-base for the clinical use of HSY to treat RIHD.

## 2. Material and Methods

### 2.1. Reagents

Rabbit polyclonal anti-TGF-*β*1, anti-Col1, anti-Col3, anti-Smad 2, anti-Smad3, anti-Smad4, and MMP14 were purchased from Gene Tex Corporation (Irvine, California, USA). Rabbit polyclonal anti-Smad7 and anti-TIMP1 were purchased from Abcam Corporation (Cambridge science park, England). Rat polyclonal anti-GAPDH and HRP were purchased from Immunoway Corporation (Plano, TX, USA). RIPA cracking liquid, PMSF, BCA protein quantitative kit, and SDS-PAGE gel kit were purchased from Solarbio Science & Technology Co. Ltd. (Beijing, China). Huangqi Shengmai Yin (HSY) was purchased from Nan Chang Ji Sheng pharmaceutical factory (Nanjing, China).

### 2.2. Experimental Animals and Group Intervention

SPF male Wistar rats (180 ± 10 g) were purchased from the Research Laboratory Animal Center of Gansu University of Traditional Chinese Medicine. Two hundred and fifty rats were randomly divided into the control group (Con), model group (Mod), low-dose HSY group (LDG, 1.6 ml/kg), medium dose HSY group (MDG, 3.1 ml/kg), and high dose HSY group (HDG, 6.2 ml/kg). HSY was given through continuous gavage for 6 weeks, with the Con and Mod rats being given equal volume of double distilled water for gavage.

### 2.3. Irradiation Procedure

Rats were given 10% chloral hydrate (3ml/kg) intraperitoneally for anesthesia prior to irradiation. The rat chest walls were exposed and the most obvious area with a heartbeat was identified so that the heart was located at the center of the irradiation field. Copper tubes were used to shield other parts of the rat from radiation. A single dose of a 25 Gy X-ray was administered to the rats, using a PRECISION X-ray (X RAD 225) (North Branford, Connecticut, USA), operating at a dose rate of 6.1 Gy/min with a source-to-skin distance of 30 cm. The exposure parameters were set to 225 KV and 13.30 mA. After irradiation, the rats were placed in cages and were maintained at 26-28°C until they woke up, in order to avoid hypothermia after anesthesia.

### 2.4. HE Staining and Myocardial Pathology Score

Hematoxylin and eosin (HE) staining was performed to reveal the morphological features of injured myocardial tissue. Pathological scoring was performed according to Rona Standards and related literature [8]. Resulting myocardial inflammatory lesions were graded as 0, 1, 2, 3, and 4 points, according to the degree of severity ranging from light to heavy. Six high-powered microscopic fields were used for scoring and the averaged was used for each slice.

### 2.5. Masson Staining and Collagen Volume Fraction (CVF)

Collagen staining was performed by Masson staining, through microscopic observation and Image Pro Plus software 6.0 was used to calculate the CVF of myocardial tissue. The formula for CVF was myocardial interstitial collagen area/total visual field area. Eight slices were taken for each group, and six high-powered fields under microscopes were scored and averaged for each slice.

### 2.6. Immunohistochemistry

Paraffin-embedded myocardial tissue was routinely dewaxed and hydrated, and the sections were rinsed with PBS and then repaired in a microwave. They were then incubated in 3% H_2_O_2_ at room temperature for 10 minutes, to block the activity of endogenous peroxidase. Afterwards, sections were incubated with the first antibody at room temperature for 2 hours and then washed with PBS. Subsequently, incubation with a polymer enhancer was performed at room temperature for 20 minutes. Then an enzyme-labeled anti-mouse/rat polymer was added and the section was incubated at room temperature for 30 minutes. After washing, fresh DAB coloring was added with incubation for 5 minutes and the samples were observed under a microscope in order to assess the degree of staining. Subsequently, hematoxylin redyeing was applied for 2 minutes, followed by hydrochloric acid alcohol differentiation and xylene transparent. The sample was then mounted on cover slips using glycerol gelatin. Image Pro Plus software 6.0 was used to calculated the average optical density (= optical density/area), which indicated the protein expression level.

### 2.7. Western Blotting

Briefly, extract cardiac tissue protein, and the protein concentrations were assessed using the Bradford assay (Roche). Proteins were separated using SDS-PAGE electrophoresis and transferred to PVDF membranes. Membranes were blocked with nonfat milk and probed with primary polyclonal antibodies. Membranes were incubated with HRP-conjugated anti-rat IgG. Antibody-bound protein bands on the immunoblot were visualized using a Chemi DOC XRS+ Gel imaging analysis system (BIO-RAD, USA).

### 2.8. Statistical Analysis

All of the data are expressed as mean ± SD. Statistical and graphical analysis was performed using the SPSS version 18.0 software package. Statistical significance was determined using a one-way ANOVA.* p* < 0.05 was considered statistically significant.

## 3. Results

### 3.1. Pathological Observation

**Figure 1:**
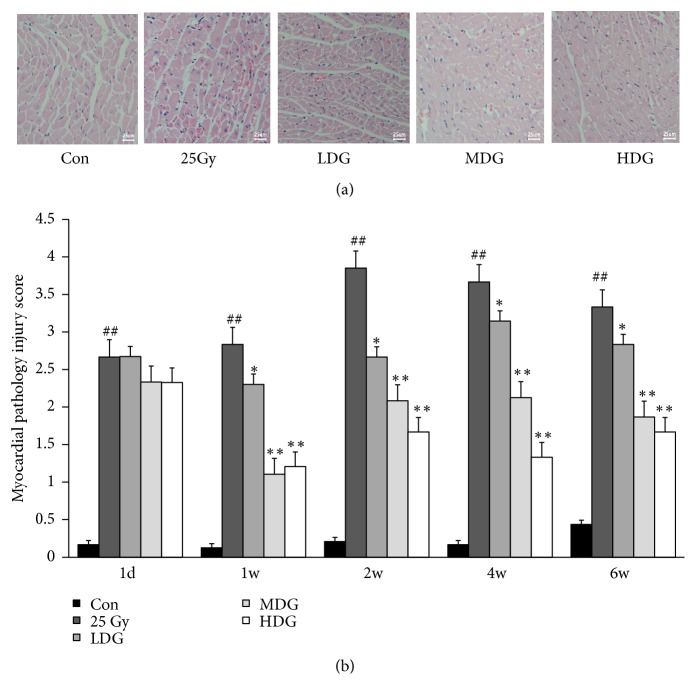
*Myocardial pathological changes in rat models*. (a) The precardiac region of rats was irradiated with 25 Gy X-rays, and HE staining was performed one week after irradiation demonstrating the pathological morphological features of injured myocardial tissue. Magnification A low dose (0.31 ml/d, LDG), medium dose (0.62 ml/d, MDG), and high dose (1.24 ml/d, HDG) of HSY were given daily to irradiated rats. HE staining was performed after a week of continuous intervention to observe the effect of HSY on the pathological injuries as a result of RIHD. Magnification ×400. (b) Pathology injury score was obtained on day 1 and weeks 1, 2, 4, and 6 after irradiation. The criteria for scoring were neatness of myocardial fibers arrangement, cell hypertrophy, degeneration, and necrosis; (2) hyperemia, edema, inflammatory cell infiltration, and connective tissue hyperplasia in myocardial interstitial; (3) congestion, edema, inflammatory cell infiltration in the endocardial, or epicardial. All lesions were rated as 1, 2, 3, and 4 points, respectively, from mild to severe, with 0 points for no lesions. ##p<0.01 versus Con; *∗*p<0.05 versus 25 Gy; *∗∗*p<0.01 versus 25 Gy.

Cardiac cells were aligned and their nuclei were stained blue and the cytoplasm was stained red in the control group (Con). At week 1 after irradiation, the disordered arrangement of myocardial fibers, capillary infiltration, and inflammatory exudation was obvious and heart valve and endocardial hyperemia edema were observed, while some myocardial cells had dissolved, and the number of fibroblasts increased (Figure 1(a)).

Our study also indicated that the myocardial pathology injury score in the Con group was the lowest (<0.5 points). At day 1 and week 1 after irradiation, the score was between 2.5 points and 3 points and increased to more than 3 points at weeks 2, 4, and 6 after irradiation which indicates that pathological damage was aggravated. Pathological injury scores of the 25 Gy group (model group, Mod) were significantly higher than that of the Con group, at corresponding time points (p<0.01) (Figure 1(b)).

HSY decreased the myocardial pathology injury score in a dose-dependent manner. Pathological inflammatory injury caused by radiation could be effectively improved in LDG, MDG, and HDG groups, and their pathological injury score in MDG and HDG group was statistically different from that of the Con group (p<0.01) (Figures 1(a) and 1(b)).

### 3.2. Collagen Quantification

**Figure 2:**
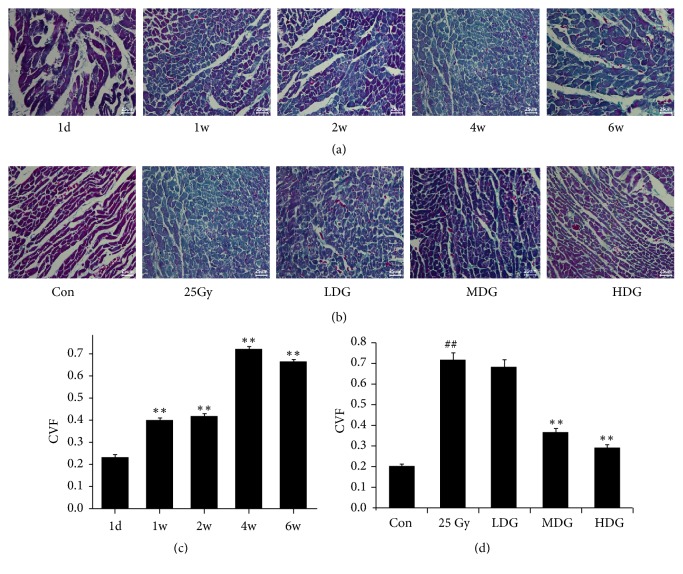
*Changes to myocardial collagen in rat models*. (a) Masson staining was performed at day 1 and weeks 1, 2, 4, and 6 after rats were irradiated with 25 Gy X-rays, revealing changes to the myocardial collagen of irradiated rats. Magnification ×400. (b) Low-dose (0.31 ml/d, LDG), medium dose (0.62 ml/d, MDG), and high dose (1.24 ml/d, HDG) of HSY were given daily to irradiated rats. Masson staining was performed after four weeks of continuous intervention to observe the effect of HSY on myocardial collagen of RIHD. Magnification ×400. (c) Collagen volume fraction (CVF) in myocardial tissue was calculated in irradiated rats. *∗∗* p < 0.01 versus 1d. (d) Collagen volume fraction (CVF) in myocardial tissue was calculated in HSY intervention rats. ^##^ p < 0.01 versus Con; *∗∗* p < 0.01 versus 25 Gy.

In Masson staining, collagen fibers were stained blue, muscle fibers and red blood cells were stained red, and nuclei were stained blue and brown. A large amount of muscle fibers and a small amount of collagen fibers was observed at day 1 after irradiation, and the CVF was 22.97%. However, the presence of collagen fibers increased significantly at weeks 1, 2, 4, and 6 after irradiation, and CVF was 39.97%, 41.83%, 72.04%, and 66.25%, respectively, which was statistically different from day 1 (p<0.01) (Figures 2(a) and 2(c)).

Our study also indicated that week 4 postirradiation seemed to be the most significant time point at which collagen fibers increased (Figure 2(c)). Based on this increase, week 4 after irradiation with 25 Gy was selected as the observation point of HSY intervention. There were almost no collagen fibers in the Con group and a large amount of collagen fibers in the Mod group; CVF increased 2.51-fold at week 4 after irradiation, compared with that of the Con group (p<0.01) (Figures 2(b) and 2(d)). In the HSY groups, collagen fibers decreased, and the CVF decreased by 4.44% (LDG group), 48.60% (MDG group) (p<0.01), and 59.16% (HDG group) (p<0.01), compared with that of the Mod group (Figures 2(b) and 2(d)).

### 3.3. Immunohistochemistry

The above results demonstrate that the pathological injury score changed significantly at week 2 after irradiation and gradually recovered at week 4 and 6 after irradiation. The highest CVF was observed at week 4 and decreased at week 6 after irradiation. Therefore, before irradiation and day 1 and weeks 1, 2, and 4 after irradiation were selected as the time points of immunohistochemical detection of fibrosis-related factors. After HSY intervention, pathological injury score and CVF were improved in a dose-dependent manner. Therefore, the immunohistochemical changes were detected in the HDG group.

**Figure 3:**
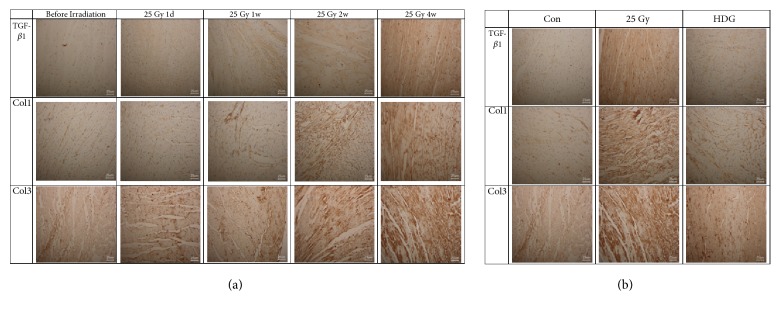
*Changes in fibrotic factors in rat injury models*. (a) Immunohistochemistry was performed at one day before irradiation and day 1 and weeks 1, 2, and 4 after irradiation to reveal changes in fibrosis-related factors TGF-*β*1, Col1, and Col3 in irradiated rats. Magnification ×400. (b) A high dose (1.24 ml/d, HDG) of HSY was given daily to be irradiated. Immunohistochemistry was performed 4w after irradiation revealed the effect of HSY on fibrosis-related factors TGF-*β*1, Col1, and Col3 in irradiated rats. Magnification ×400.

The results showed that fibrosis factors Col1 and Col3 were expressed in cytoplasm and that TGF-*β*1 is mainly expressed in the cytoplasm and partially expressed in the nucleus (Figure 3). At day 1 and weeks 1, 2, and 4 after irradiation the expression of TGF-*β*1 increased by 12.3%, 25.7%, 37.5%, and 46.6%, respectively; Col1 increased by 16.0%, 21.8%, 29.4%, and 41.6%; Col3 increased by 6.7%, 22.5%, 63.2%, and 80.4%, respectively, compared with that prior to irradiation (p < 0.05) (Figure 3; Table 1). The above results indicated that the expression of fibrosis factor increased with time after irradiation, so week 4 after irradiation also seemed to be the most significant time point for the increase of fibrosis factors. Therefore week 4 after irradiation was selected as the observation point of HSY intervention. In the Mod group, the TGF-*β*1, Col1, and Col3, increased by 93.2%, 27.5%, and 22.7% (p<0.01), respectively, compared with that of the Con group, while these factors decreased by 29.6%, 14.8%, and 13.4% in the HDG group, compared with that of the Mod group (p<0.01) (Figure 3; Table 2).

### 3.4. Western Blotting

The above immunohistochemical results preliminarily showed that HSY could regulate radiation-induced increases in the expression of fibrosis-related factors. To further confirm these results, western blotting detection was performed.

**Figure 4:**
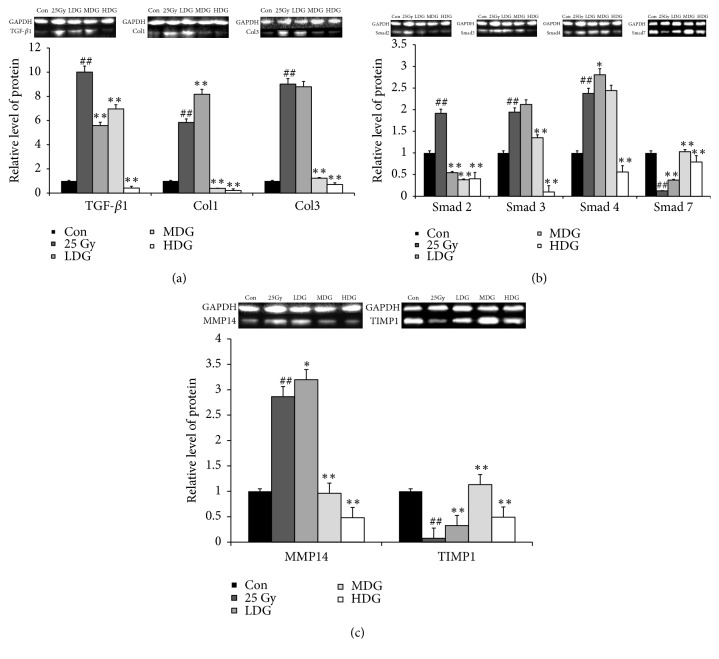
*Regulation of HSY on TGF-β1/Smads signaling pathway and MMPs system in irradiated rats*. A low dose (0.31 ml/d, LDG), medium dose (0.62 ml/d, MDG), and high dose (1.24 ml/d, HDG) of HSY were given daily to irradiated rats. (a) The protein expression of TGF-*β*1, Col1, and Col3 in irradiated rats was analyzed with western blotting. (b) The protein expression of Smad 2, Smad 3, Smad 4, and Smad 7 in irradiated rats was analyzed with western blotting. (c) The protein expression of MMP14 and TIMP1 in irradiated rats was analyzed with western blotting. The average fluorescence intensity of each protein band is shown. All data are representative of three independent experiments. ^##^p<0.01 versus Con; *∗*p<0.05 versus 25 Gy; *∗∗*p<0.01 versus 25 Gy.

Relative protein levels of fibrosis-related factors TGF-*β*1, Col1, Col3, MMP14, Smad 2, Smad 3, and Smad 4 increased 9.0-fold, 4.8-fold, 8.0-fold, and 1.86-fold, 91.5%, 94.4%, and 137.5% at four weeks after irradiation, compared with that of the Con group (p<0.01) (Figures 4(a), 4(b), and 4(c)). Protein expression of TIMP1 and Smad 7 decreased by 92.4% and 87.6%, compared with that of the Con group (p<0.01) (Figures 4(b) and 4(c)).

Protein expression of TGF-*β*1 decreased by 44.2 (LDG), 30.5% (MDG), and 95.8% (HDG), Col1 decreased by 93.3% (MDG) and 96.3% (HDG), Col3 decreased by 2.6% (LDG), 86.3% (MDG), and 92.1% (HDG), Smad 2 decreased by 71.5% (LDG), 80.2% (MDG), and 78.9%(HDG), Smad 3 decreased by 30.5% (MDG) and 95.0% (HDG), Smad 4 decreased by 76.4% (HDG), and MMP14 decreased by 66.4% (MDG) and 83.2% (HDG), compared with that of the Mod group (p<0.01) (Figures 4(a), 4(b), and 4(c)). Protein expression of Smad 7 increased 2.0-fold (LDG), 7.3-fold (MDG), and 5.4-fold (HDG), and TIMP1 increased 3.3-fold (LDG), 13.9-fold (MDG), and 5.5-fold (HDG), compared with that of the Mod group (p<0.01) (Figures 4(b) and 4(c)).

## 4. Discussion

Model animals used in previous RIHD research include rats [8], mice [9], dogs [10], rabbits [11], and rhesus monkeys [12]. Experimental zoology rats are most suitable for the study of cardiomyopathy [13]. The pathological changes in heart injury of rats include myocardial degeneration and fibrosis, which is consistent with the characteristics of RICF. Therefore, we selected rats as model animals to carry out the RICF experimentation.

In studies of RIHD, the radiation doses in animal models differ greatly. The radiation dose reported in literature included several Gy values [12], but most of them were above 10 Gy, with the highest being 50 Gy [14] and 60 Gy [15]. Many studies show that the most common radiation dose in modeling was 15 Gy, 18 Gy, 20 Gy, 25 Gy, and 30 Gy [16]. The large difference in irradiation dose for modeling is related to the size of the irradiation field. Many tissues and organs of animals are damaged by radiation during whole body irradiation; the animals are unable to tolerate this and die. However, when animals received local irradiation only in the precardiac region, their whole body was less affected by the radiation, and they were able to tolerate a large dose of irradiation [11, 17]. Considering that the heart is a radiation-insensitive organ, this high dose of local irradiation to the heart has more significant damage to the heart and is more suitable for in animal RICF research. In the preliminary experiments, it was found that when the radiation dose was increased to 25 Gy, it could cause severe fibrosis injury and structural damage to the heart. Therefore, it is considered that an X-ray dose of at least 25 Gy to the precardiac area was necessary for the construction of RICF conditions in a rat model.

A literature review made clear that the most common experimental methods used in RIHD basic research included pathological HE and Masson staining, immunohistochemistry, PCR, and western blotting [16]. The most common indicators used are histopathological observation, myocardial enzyme, inflammatory, and fibrosis-related cytokine detection [16, 18]. In this study, most of these experimental methods and detection indexes were used to evaluate the RICF model established through local irradiation of 25 Gy to the precardiac region.

Pathological morphology observation is the preferred method for RIHD animal experiments [16]. In this study, the myocardial structure of the Con group was relatively normal, and the myocardial pathology injury score was relatively low. However, the score of the Mod group increased along with the increase of time after irradiation, indicating that pathological injury persisted and increased despite the end of irradiation, which may be related to the delayed effect of radiation injury. Similar trends in CVF suggest a progression of fibrosis over time. These results demonstrated that 25 Gy X-ray could establish animal models of acute and delayed radiation injuries.

Fibrosis is the terminal stage in the development of RIHD and the main form of delayed radiation injury, which can be seen in various clinical types of RIHD. RICF is a progressive fibrosis process of collagen synthesis and degradation imbalance that is mainly manifested as an increase in CFs and myocardial collagen synthesis [4]. It can be clearly argued that radioactive fibrosis is characterized by an upregulated expression of TGF-*β*1 [19, 20]. In vitro experiments confirmed that mRNA expression of TGF-*β*1 was significantly elevated in irradiated rat myocardial cells, heart endothelial cells, and CFs [21]. TGF-*β*1 can bind to its receptor, which stimulates epithelial hyperplasia, CFs proliferation, and collagen deposition of irradiated tissues and participates in the entire process of fibrosis [18]. Activation of TGF-*β*1 is associated with the ROS produced by radiation. Oxidative stress is extremely efficient in the TGF-*β*1 activation [22]. ROS produced by radiation could induce direct DNA damage [23] and an inflammation [24]. DNA damage and inflammation could activate TGF-*β*1 [25]. Our previous research showed that administration of sodium tanshinone IIA sulfonate [26] and astragalus saponin [27] could inhibit the expression of TGF-*β*1 and attenuate fibrosis damage effect in irradiated CFs, and this antifibrosis effect may be closely related to their antioxidant action.

At present, TGF-*β*1 is considered to be one of the cytokines that play a key role in the process of fibrosis. In the early stage of radioactive injury, the expression of TGF-*β*1 mRNA is more obvious than the histological changes, and the average expression of TGF-*β*1 mRNA was elevated earlier than those of Col1 genes [28], so it can early indicate the occurrence of tissue fibrosis. TGF-*β*1 is involved not only in the onset but also in the development of radiation fibrosis [29]. The mRNA expression of TGF-*β*1 was positively correlated to the proportion of collagen fibers in the irradiated rat hearts. Interference with the TGF-*β*1 pathway has also been proved to protect against fibrosis formation [30] and therefore downregulated the expression of TGF-*β*1 mRNA in rats to modify the development of RIHD [31]. In this study, TGF-*β*1 and myocardial collagen Col1/Col3 were used as pathological molecular indicators to reflect the pathological process of fibrosis in RIHD; the expression of these molecules showed a gradually increasing trend, which is consistent with the changes of CVF in Masson staining, suggesting that fibrosis pathology continued to progress with time after irradiation. Another study showed that rats were irradiated with a single dose≦20 Gy; mRNA level of TGF-*β*1 increased at days 1 and 12 and then returned to control levels by 1 month. But at 25 Gy, there was a persistent elevation of TGF-*β*1 mRNA for >6 months, and mRNA for Col1 and Col3 also increased [32]. These experimental results also confirmed the reliability of the 25 Gy single irradiation method for the construction of progressive RICF model.

TGF-*β* signaling molecules mediate a wide variety of cellular functions. These functions are inseparable from integrins. Integrins could mediate cell adhesion, differentiation, migration, proliferation, and matrix remodeling through the mutual transformation of intracellular and extracellular signals [33]. Integrin-mediated TGF-*β* activation seems to be possible in a Smads-dependent or protease-dependent manner.

The activation of TGF-*β* signaling pathway begins with TGF-*β* binding to serine/threonine kinase receptor complex, which consists of TGF-*β*r1 and TGF-*β*r2, subsequently leading to the recruitment and phosphorylation of the intracellular effector proteins Smad2/Smad3, and then phosphorylated Smad2/Smad3 binds to Smad4 and translocates to the nucleus to initiate gene expression [34]. Smads have a relatively weak affinity for DNA [35] and need to use other factors to form a robust complex with high affinity and specificity to cognate DNA sequences. These include many profibrotic genes, such as Col1a2 and the TGF-*β*1 gene itself [36]. The TGF-*β* signaling pathway could be negatively regulated by inhibitory Smads, including Smad6 and Smad7 [37]. A study strongly suggests that downregulation of Smad7 leads to an amplification of TGF-*β* signaling, which contributes to the progression of inflammation and fibrosis [38]. Our previous study has shown that TGF-*β*1, Smad2, Smad3, and Smad4 were upregulated in CFs when treated with 1 Gy X-ray. In addition, Smad7 was downregulated when treated with 1 Gy X-ray [7, 27]. This animal experiment further confirmed that the relative expressions of Smad 2, Smad 3, and Smad 4 were increased when treated with 25 Gy X-ray, while the expression of Smad 7 was significantly decreased. Our results suggested that irradiation seemed to promote the activation of TGF-*β* signaling pathway and meanwhile suppress its negative regulation and therefore promote the fibrosis development. This experiment also found that HSY could improve RICF by reversing the above Smads expression. Similar to our study, Glycyrrhetinic acid could protect against radiation-induced lung injury. Its protective effect may be also associated with inhibition of the TGF-*β*1/Smads signaling pathway [39]. These results proved that the TGF-*β*1/Smads pathway is involved in radioactive fibrosis. In addition, radiation could promote endothelial-to-mesenchymal transition (EndoMT) which relates to organ fibrosis; TGF-*β*1/Smads pathway activation may be involved in EndoMT [40]. And another study has shown that connective tissue growth factor (CTGF, recently renamed CCN2 [41]) transactivation by TGF-*β* may be also associated with Smad pathway [42]. In summary, TGF-*β*1/Smads pathway plays a complex role in radioactive fibrosis, which requires further research.

MMPs, a family of Zn-dependent endopeptidases, degrade all kinds of extracellular matrix (ECM) proteins. In fibrosis pathology, MMPs and TIMPs could involve in matrix regulation; for example, MMPs can play a role in collagen matrix degradation [43]. In addition, MMPs participate in the activation of TGF-*β*. Protease-dependent TGF-*β* activation needs to recruit MMP14, which then releases TGF-*β* by proteolytic cleavage[44], and TGF-*β*1 has been shown to be a potent transcriptional activator of TIMP1 [45]. In TGF-*β*1 transgenic mice, the expression of TIMPs was upregulated, thereby decreasing possibly the ECM degradation leading to lung fibrosis [46]. In contrast, the downregulated expression of TGF-*β*1 and TIMP accounts for the elimination of collagenase activity inhibition and the subsequent digestion of excess ECM deposition, as well as radioactive fibrosis reversibility in vivo [47]. Our previous study showed that MMP14, MMP3, and MMP8 of remodeling enzymes were upregulated under treatment with 1 Gy X-rays, whereas TIMP1 was downregulated. These findings suggested that the TGF-*β* activation mediated by MMP14 was strengthened and the fibrosis process was started [7, 27]. This animal experiment showed that protein expression of MMP14 increased 1.86-fold after 25 Gy X-ray irradiation, whereas TIMP1 decreased by 92.4%, which was consistent with the previous experimental results, indicating that the balance between TIMPs and MMPs was disturbed by radiation, protease-dependent TGF-*β* activation was initiated, and the imbalance of collagen synthesis and degradation resulted in fibrosis effect. This animal experiment also showed that HSY could attenuate RICF by reversing the radiation effect on expression of MMP14 and TIMP1. This regulatory effect of HSY, in turn, confirmed that MMPs/TIMP system involved in RICF.

In traditional Chinese medicine (TCM), radiation is considered to be the evil of heat and poison, and irradiation to the heart causes toxic heat and blood stasis syndrome, deficiency of both Qi and Yin, Qi stagness and blood stasis, and chronic diseases transforming to collaterals, which leads to fibrosis. In addition, Chinese medicine believes that the heart governs the blood. The main pathogenesis of cardiotoxicity induced by radiotherapy is the deficiency of both Qi and Yin and the deficiency of Qi and blood. TCM can effectively adjust heart function through the intervention of invigoration of the heart-Qi and nourishing blood and activating blood. Therefore, TCM intervention should focus on “supplementing Qi and nourishing Yin”, and the prevention and maintenance of vital Qi are the key.

Shengmai Yin is mainly used to treat coronary heart disease and myocardial infarction characterized by palpitation and dyspnea, which is caused by a deficiency of both Qi and Yin. In the clinical literature it has been reported that Shengmai injection can effectively protect the heart and reduce the incidence of serum myocardial enzyme abnormality during radiotherapy of thoracic tumors [48]. In addition, Shengmai Yin has a clear effect in antiradiation-induced pneumonia and pulmonary fibrosis [49]. Shengmai Yin could also improve ventricular remodeling and myocardial fibrosis through its antioxidant activity and by inhibiting the overexpression of CTGF [50]. It may also reverse ventricular remodeling and improve cardiac function by inhibiting NF-*κ*B [51].

Astragalus is a traditional Chinese medicine, used to strengthen the heart, dilate the coronary blood vessels, resist oxidation, anti-inflammatory, and antifibrotic, and enhance immunity. Our previous study demonstrated that astragalus can resist X-ray induced fibrosis injury of CFs by regulating TGF-*β*1/Smads signaling pathway and mediating imbalance between TIMPs and MMPs [7, 27]. This mediating effect of astragalus on MMPs/TIMPs was also confirmed in the study on rat pulmonary fibrosis [52]. There have also been clinical reports that astragalus injection can reduce the incidence of the cardiac inflammatory response during radiotherapy of chest tumors and reduce the incidence of ECG abnormalities, as well as cardiac discomfort symptoms such as chest distress, palpitation, and chest pain [53].

Therefore, based on the TCM theory that irradiation to the heart causes deficiency of both Qi and Yin, Qi stagnation, and blood stasis, this study assumed that TCM intervention should focus on supplementing Qi and nourishing Yin and nourishing blood and replenishing Yin, so HSY is used in a RIHD rat model. In the prescription of HSY, astragalus can invigorate Qi, activate Qi, and promote blood flow; ginseng can enhance the yang, restore pulse and control prostration, invigorate the spleen to benefit the lung, and generate fluid and tranquilization; liriope can nourish Yin to clear away heat and generate fluid, moisten the lung, and relieve the heart; and* Schisandra chinensis* can converge heart-Qi. The whole prescription plays a role in supplementing Qi, nourishing Yin, and nourishing Yin and blood. The results of this study showed that HSY could reduce cardiac pathological injury score after irradiation in a dose-dependent manner. At the same time, CVF was reduced, and the high expression of fibrotic molecules TGF-*β*1, Col1, and Col3 was downregulated, thus inhibiting the pathological process of fibrosis. In addition, this study also found that HSY could inhibit the upregulated expression of Smad2, Smad3, Smad4, and MMP14 caused by X-ray and reverse the downregulated expression of Smad7 and TIMP1 caused by X-ray. Therefore, it can be concluded that the TGF-*β*1/Smads signaling pathway and MMPs system are the possible pharmacological mechanisms of HSY to improve RICF.

In conclusion, the TGF-*β*1/Smads signaling pathway and MMPs system were involved in the pathological process of RICF. A medium and high dose of HSY could effectively improve RICF by regulating the above signaling molecules. The present findings strongly support the interpretation that HSY may be beneficial in protecting myocardium against radiation-induced heart damage.

## Figures and Tables

**Table 1:** The average optical density of fibrotic factors in rat models.

	Before Irradiation	25 Gy 1d	25 Gy 1w	25 Gy 2w	25 Gy 4w
TGF-*β*1	0.253±0.011	0.284±0.014*∗*	0.318±0.006*∗∗*	0.348±0.007*∗∗*	0.371±0.018*∗∗*
Col1	0.262±0.014	0.304±0.015*∗∗*	0.319±0.004*∗∗*	0.339±0.015*∗∗*	0.371±0.005*∗∗*
Col3	0.285±0.011	0. 304±0.017	0.349±0.019*∗∗*	0.465±0.027*∗∗*	0.514±0.024*∗∗*

The average optical density from immunohistochemistry sectioning was calculated, which indicated preliminarily fibrotic factor expression levels. *∗*p<0.05 versus before irradiation, *∗∗*p<0.01 versus before irradiation.

**Table 2:** The average optical density of fibrotic factors in the HSY intervention group.

	Con	25 Gy	HDG
TGF-*β*1	0.192±0.023	0.371±0.018^##^	0.261±0.036*∗∗*
Col1	0.291±0.009	0.371±0.005^##^	0.316±0.012*∗∗*
Col3	0.419±0.025	0.514±0.024^##^	0.445±0.006*∗∗*

The average optical density from immunohistochemistry sectioning was calculated which indicated preliminarily the effect of HSY on fibrotic factors expression level. ^##^p<0.01 versus Con; *∗∗*p<0.01 versus 25 Gy.

## Data Availability

The data used to support the findings of this study are available from the corresponding author upon request.
